# A Big Data Platform for Real Time Analysis of Signs of Depression in Social Media

**DOI:** 10.3390/ijerph17134752

**Published:** 2020-07-01

**Authors:** Rodrigo Martínez-Castaño, Juan C. Pichel, David E. Losada

**Affiliations:** Centro Singular de Investigación en Tecnoloxías Intelixentes (CiTIUS), Universidade de Santiago de Compostela, 15705 Santiago de Compostela, Spain; juancarlos.pichel@usc.es

**Keywords:** Social Media, text mining, depression, public health surveillance, stream processing, real-time processing

## Abstract

In this paper we propose a scalable platform for real-time processing of Social Media data. The platform ingests huge amounts of contents, such as Social Media posts or comments, and can support Public Health surveillance tasks. The processing and analytical needs of multiple screening tasks can easily be handled by incorporating user-defined *execution graphs*. The design is modular and supports different processing elements, such as crawlers to extract relevant contents or classifiers to categorise Social Media. We describe here an implementation of a use case built on the platform that monitors Social Media users and detects early signs of depression.

## 1. Introduction

In recent years, the impact of Social Media (SM) on public health aspects has received increasingly more attention. This paper focuses on the technological challenges related to the construction of effective and scalable systems able to support Social Media Intelligence tasks. Although our approach is general and the system can assist different surveillance tasks, we concentrate here on showing its potential for early detecting signs of depression.

Computational methods for screening Social Media and supporting mental state assessments is a thriving area of research. Many SM users publicly share their feelings and emotions. The availability of large samples of user-generated contents provides a unique opportunity for designing health surveillance tools that proactively analyse signs of mental disorders such as anxiety or depression. Such online screening tools are valuable and might act in the future as a complement to more standard assessment strategies.

The impact and prevalence of common mental disorders, such as depression, is severe. In January 2020, the World Health Organisation (WHO) estimated that, globally, more than 264 million people of all ages suffer from depression (https://www.who.int/news-room/fact-sheets/detail/depression). The burden of depression is on the rise and it is a major cause of disability worldwide. Although there are effective treatments for depression, a large percentage of people receive no treatment for their disorder. WHO states that inaccurate assessment is a barrier to effective care. Most computer-based analytical tools focus on structured clinical data [1]. We follow here a different route and claim that innovative online screening methods can advance in early detecting cases of depression.

Lack of early detection of depression might lead to serious health consequences, such as disability, psychotic episodes and even suicide. It is crucial to identify the onset of mental disorders at early stages and develop appropriate psychological intervention strategies.

SM data provide substantive evidence about the behaviour and mental state of people. Popular SM platforms, such as Twitter or Reddit, have millions of users worldwide and receive huge amounts of publications per minute. These vast repositories of user-generated text give us an opportunity to design new SM tools that analyse texts and extract signs or symptoms related to the onset of depression. As argued by [2], SM analytics is promising in the mental health domain because popular SM sites provide access to naturalistic, first person accounts of people’s behaviour, thoughts, and feelings. SM evidence can therefore supply valuable indicators related to the emotional well-being of the individuals.

There is evidence that people increasingly turn to online platforms to share their moods, thoughts and feelings [3,4,5,6,7,8]. There are a variety of reasons for individuals to publicly disclose information about their personal concerns and mental state [3]. This includes seeking or offering support, and fighting the stigma of mental disorders.

Automatic extraction of well-known symptoms of depression (e.g., guilt, sadness or pessimism) might be done from SM data, leading to new forms for the screening of this mental disorder. The words or expressions that people use can be revelatory of their psychological states. A large stream of studies have engaged in the psychological aspects of natural language use [9,10,11]. Explicit and implicit factors, including subtleties in the linguistic styles of the individuals, can link text to a wide array of dimensions, such as personality, psychological state, and social and situational fluctuations, just to name a few. For example, textual (or content-based) features can act as markers of personality [12,13], psychological state [9,14], personal values [15], or can even inform about drug effects [16].

The use of SM data to design novel intelligent tools for public health surveillance is therefore a real possibility. By leveraging online data we can assist health experts (or other stakeholders involved in public health surveillance) in screening communities or specific individuals at-risk. Large-scale analysis of SM data offers low-cost and non-intrusive methods that, following proper ethical guidelines, could be employed by health institutions [17]. However, the large volume of SM users and contents demands new technological solutions that support real-time text mining of health-related pieces of evidence. This is precisely the main aim of this paper.

There are many technologies designed for massive data processing. Some of them are open source and, thus, research teams or institutions can freely adopt them for public health surveillance. However, as shown in this paper, they have limited scalability. We contribute here with a library that naturally eases the development of applications that process massive amounts of stream data. SM sites are environments where millions of users produce contents in real-time. Our library can support the construction of new SM intelligence tools to analyse the onset of psychological problems. As a matter of fact, we present here a use case of the library where we built a depression analysis tool. The tool exploits existing labelled depression data [18] for monitoring in real-time the appearance of signs related to depression. Related to this, we build an automatic classifier that scans the thread of posts and comments sent by users to a popular SM platform and predicts the occurrence of signs of depression.

The rest of the paper is structured as follows. Section 2 puts this research in context by briefly introducing stream processing technologies and their main characteristics. Section 3 presents our Python library that facilitates the construction of execution graphs. Section 4 develops a use case where we build an early risk detection system that is connected to SM data and alerts about signs of depression. Section 5 explains how systems developed using other programming languages can be connected to our execution graph. In Section 6, we compare our solution with other Big Data technologies. Finally, Section 8 presents the main conclusions of this study and suggests lines of future work.

## 2. Stream Processing Technologies

Existing solutions for massive data processing work with clusters of computers and typically focus on *horizontal scalability*. This means that more tasks can be performed by incorporating more similar nodes to the cluster. For example, in SM, if we are processing users and the cluster becomes exhausted then we can add new nodes to the cluster, resulting in a proportional increase of the number of users that can be monitored. An important advantage of this approach is that the new nodes do not need to be more powerful than the existing ones. Horizontal scalability requires that the data can be partitioned. Different data items are processed by different processes that are distributed on the cluster’s nodes and, therefore, run in parallel. At some point, partial results can be merged or aggregated to generate final results. Many data processing frameworks follow this paradigm now, democratising its use through commodity hardware.

Batch and stream are the two main classes of processing technologies. When using batch, the results are obtained after processing a full batch of data. An *execution graph* is responsible for performing the computation. The graph defines the path to be followed by the data and it is composed of a series of nodes or stages (e.g., filters or transformers that are applied to the data items). Each computer in a cluster receives partitions of the input data and executes one or more instances of the entire graph. When there is a sequential dependency among stages, individual results are delayed since the subsequent stages are not started until the previous ones are completed. With stream processing, the stages of an execution graph are not dependant of a global instance of it and the data flow through all the stages without locks until all the items are processed in the previous ones. Such an approach is appropriate for designing real-time applications because independent results are obtained as soon as data items complete their path through the execution graph. Since the processing stages are independent and there aren’t fixed partitions of the input data, stages can be scaled out by launching new instances even when the application is already running. Furthermore, given new data, the execution graph does not need to be relaunched since the instances of each stage of the execution graph are constantly waiting for new input data.

Most applications need to define processing stages whose computational requirements show a high variance. For example, a word filtering stage (e.g., remove common words from an online publication) might be substantially quicker than a summarisation stage (produce an abstractive or extractive summary of the publication). The wide range of computational needs can naturally be handled by assigning more resources to heavier modules and, thus, balancing the execution times of different stages.

In SM analytics, multiple use cases require Information Retrieval and Text Mining techniques and can take advantage of stream processing. For example, monitoring user data and analysing the onset of signs of depression for Public Health surveillance needs to work in real time. In this way, the resulting automatic screening tools can quickly alert about users at risk and take appropriate preventive measures. Furthermore, this type of SM intelligence tools have easily identifiable stages, associated to different processing steps. Data should follow one or several paths through the execution graph because a module could output data to multiple modules (or receive input from multiple modules). Two common stages are data extraction, which often runs at the beginning of the execution graph, and storage, which often runs at the end of the execution graph and is responsible for storing results. By designing an execution graph that incorporates the proper stages, we can implement multiple text analytics and mining processes on stream data. For example, we can support Real-Time Filtering (with a filtering stage that removes non-relevant contents), Real-Time Summarisation (with proper filtering and summarisation stages), Real-Time Clustering (grouping users or contents based on similarity and assigning them to clusters in real-time), Real-Time User Classification (categorising users based on predefined classes), and Real-Time Trend Detection (detecting new events), just to name a few.

**Figure 1:**
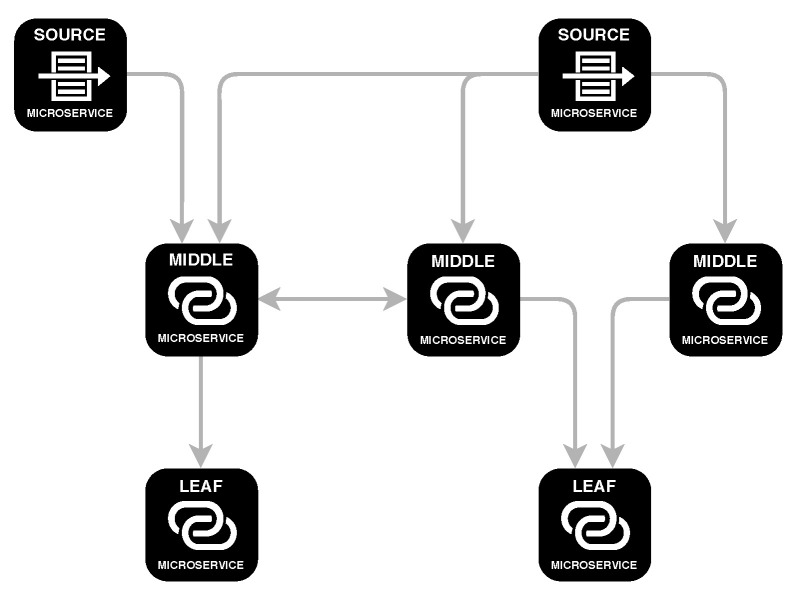
Example of an execution graph in CATENAE.

In this paper, we make two main contributions. First, we present CATENAE (https://github.com/catenae), a publicly available framework that facilitates the design of real-time applications that need to process stream data, such as SM publications. In Figure 1 we sketch an example of an execution graph, which is represented in the form of a directed graph. The nodes in the graph represent elements of filtering or data transformation, while the edges represent how data flows from one node to another. The example shows that a node can be connected to several nodes (the output of one node connected to several nodes, or the input of one node connected to several nodes). It is possible to create cycles because the data can be sent to a previous stage. This cyclical feature is useful, for example, when a temporal condition is not met and we need to redirect the data to a previous stage. A second contribution of this study is to build a user classifier that categorises SM users and estimates the presence of signs of depression. This classifier can be naturally incorporated as a processing element in CATENAE and this incorporation leads to a use case of the platform to support public health surveillance.

## 3. Catenae Topologies

An important component of execution graphs is the way in which nodes communicate with each other. In our library, communication is handled by queues of messages that are managed by Apache Kafka [19]. As argued above, if the workload of some processing element or node becomes too high then the node can be replicated. This ability to create multiple instances prevents the execution graph from experiencing bottlenecks. Batch processing solutions assign hardware resources to the entire execution graph, while CATENAE assigns resources to node instances as required.

In the following, the nodes or stages of the graph will be referred to as microservices. In CATENAE, microservices are tiny and loosely couple scripts written in Python and packaged into Docker containers. This processing technology can quickly scale up or down by launching or stopping instances of the microservices.

**Figure 2:**
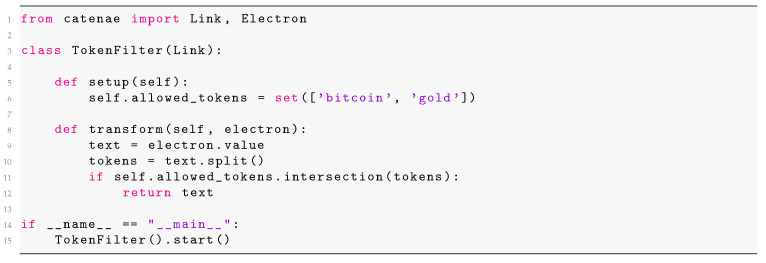
Implementation of a middle microservice (token filter).

There are three main classes of microservices in CATENAE:

- **Source microservices**. These microservices are responsible for collecting data from external sources, such as API services, access to databases or web crawlers. For example, connecting the execution graph to a stream source, such as a SM site, requires to define a source microservice that reads from the SM’s API or constantly crawls the site. This class of microservices does not receive data from other nodes and, thus, does not get input from Kafka.
- **Middle microservices**. These microservices receive data from other modules, make some sort of transformation or filtering, and emit data to other modules. For example, filters or classifiers constantly consume and emit data and can be developed in a very simple way. To further prove this point, Figure 2 shows how to code a simple filter in CATENAE. The Python class works from a set of allowed tokens (e.g., pre-defined keywords) and only those texts that have some of the allowed tokens are passed to the next stage.
- **Leaf microservices**. These microservices consume data from other nodes but do not send data back to the execution graph. They are responsible for storing results (for example, by writing on a database or generating an output file). When monitoring SM data, an instance of the execution graph can contain a leaf node that generates alerts about users “at-risk” (based on the output of a user classifier).

The microservices run into Docker containers and the administrator of the execution graph can scale it up by launching more containers (this also avoids the GIL problem, which happens when multiple threads cannot execute bytecodes of Python at once: https://wiki.python.org/moin/GlobalInterpreterLock). In our case, each instance has its own interpreter of CPython and, thus, the platform is free from this problem.). Furthermore, CPU or memory resources can be restricted for each instance of a microservice. Another advantage is that modules are isolated and can have their own dependencies. This prevents execution problems derived from lack of dependency or conflicts between the nodes of the cluster. The microservices can even have different versions of Python, as long as the shared data between microservices remain compatible.

The proposed platform supports multiple inputs for a given node. There are two possible ways to handle this case:

- **Parity**, where the microservices receive data indistinctly from their inputs.
- **Exponential**, where the microservices apply time windows to ingest data from their inputs. Each input is assigned a fraction of the time. This fraction grows exponentially with the input priority.

The microservices can output any Python object. Python objects are automatically serialised and compressed (and, after reception at the next microservice, objects are automatically deserialised).

It is possible to load external resources during the initialisation of a given microservice (for example, load a Python dumped object) with CATENAE. In addition, it supports Aerospike [20], a key-value distributed store, that can be used to quickly load data during the initialisation stages. Aerospike utilises RAM memory or SSD disks to store data and supports disk persistence when using memory.

## 4. Use Case: Real Time Analysis of Signs of Depression

In this section we explain how to employ the Big Data technologies described above for designing and deploying a SM intelligence tool that supports public health surveillance. More specifically, we designed, implemented and deployed an automatic screening tool that ingests SM contents in real-time and estimates the appearance of symptoms related to depression.

The SM source selected was Reddit, which is currently ranked in Alexa [21] as the 6th website with more traffic in the United States and the United Kingdom, and the 20th globally. It has more than 430 million monthly active users, more than 130,000 active communities (https://redditinc.com/press) and an average of 21 billion screenviews per month. According to our preliminary tests (https://github.com/brunneis/reddit-crawler), there are at least 2.9 million posts a day with peaks of 76 posts per second.

Reddit offers quite open data policies in comparison with other SM sites. It has thousands of active communities (also known as *subreddits*) and some of them are oriented to mental health support. These features make Reddit an ideal candidate for our case study.

First, we describe the crawling technology developed on our platform in order to ingest publications made in Reddit. Next, we present our classification strategy to process user-generated data and estimate the onset of depression. Then, we proceed to explain the topologies needed to deploy this user-based classification technology. Finally, we present the resulting online screening tool.

### 4.1. Social Media Crawler

In Reddit, user submissions are in the form of posts or comments that can contain text, pictures or links. Reddit users, known as *redditors*, can vote for or against any submitted content. This SM site is organised into subreddits, which are communities focused on specific themes.

**Figure 3:**
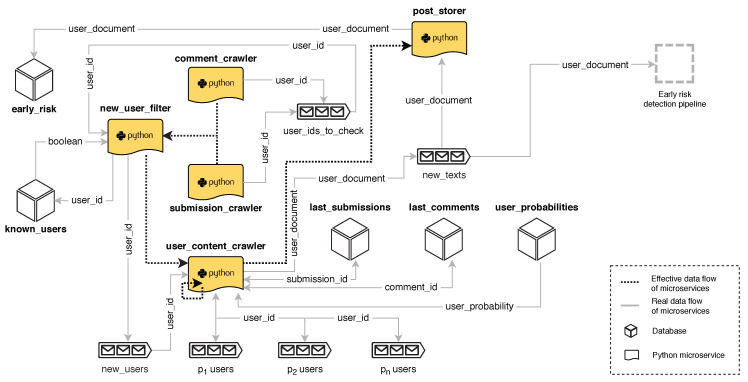
Diagram of the Social Media crawler.

A key goal when monitoring users in SM is to maximise the number of users and contents that can be analysed. To this aim, we need to constantly collect new user submissions (posts or comments) and, thus, we developed a web crawler to extract contents published in Reddit. We followed standard Robot Exclusion Protocol guidelines and respected the constraints imposed by Reddit in its robots.txt file. Our crawler uses CATENAE and incorporates multiple horizontally-scalable microservices. The architecture is shown in Figure 3. The main elements of the crawler are:

- **Crawlers of posts and comments**. These crawlers constantly extract new posts and comments in order to discover author’s usernames.
- **User filter**. Usernames are filtered to avoid repetitions. An efficient memory-based storage system, Aerospike, is employed to support this filtering task. The users who pass the filter are sent to a queue of new users. The queue of users to be crawled is organised as a priority queue. In this way, usernames can be ordered following criteria, such as user’s activity. At this point, we can also analyse the *partial information available* from the user (e.g., last extracted submission, obtained when the username was discovered) and insert the username in the queue using a priority score that estimates how probable is that the user shows signs of depression.
- **Crawlers of user’s contents**. All textual items (posts or comments) submitted by the users in the queue are extracted. In Reddit, collecting all the posts made by a given user requires to make *n* calls to the Reddit web server (where *n* is the number of submissions made by the user and a maximum of 100 requests are allowed), while collecting the comments submitted by a user requires only a single call. An output queue stores the extracted textual items.
- **Content storer**. It stores the textual contents in a document-oriented database. These texts will later be fed to the early prediction modules (see Section 4.3).

Usernames can be quickly extracted because a single instance that monitors the appearance of new contents in Reddit is able to discover multiple usernames. The main bottleneck is the User Content Crawler, which needs to make multiple calls to extract all user’s data. This is the only module in our execution graph that requires a large number of instances.

### 4.2. Depression Classification

The Social Media crawler described above constantly extracts SM users and permits to keep an updated representation of each user. New submitted contents are quickly detected and stored. This *incremental representation* of the users can be thought of as a sequential accumulation device that permanently learns about each user. Although this technology has the potential to support multiple SM intelligence tasks, we focus here on how to employ it to support the detection of early signs of depression.

There is substantial evidence in the literature that shows that the words people use are revelatory of their psychological condition. In particular, depression has been associated with distinctive linguistic patterns [4,9,10,22,23,24]. With the increasing use of Social Media, this type of patterns can be extracted and analysed using online data.

Language use is a powerful indicator of multiple aspects of the individuals, such as their social status, their personality, or their emotions. In terms of mental health, the interactions between natural language use and different psychological disorders has received increasing attention. For instance, Pennebaker [9,10] showed that depression is associated with an elevated use of first person pronouns. Text analytics techniques can therefore be useful to estimate the onset of depression. Indicators such as an increasing use of words related to negative emotions, words related to feelings of guilt, or words related to the self can be valuable signs of risk. Traditional instruments, such as Beck’s Depression Inventory [25], measure the severity of depression based on multiple-choice self-report questions (composed of items relating to symptoms of depression such as fatigue, guilt, or weight loss). The application of Big Data techniques to massive amounts of SM data can also provide valuable insights about this type of symptoms and, following proper ethical guidelines, new depression screening tools could be designed.

With supervised learning technology we can build a depression language classifier, an automatic tool able to distinguish between the language of depressed and non-depressed individuals. However, such construction requires a sample of labelled data (examples of text written by a depressed group and examples of text written by a control group). Traditional research studies on depression and language have been confined to clinical environments (for example analysing written essays or spontaneous speech) and data cannot be redistributed. On the other hand, some studies on depression and SM [4,22,23,24] suggested innovative methods to gather SM contents published by individuals diagnosed with depression. However, these teams often worked with SM sources whose contents cannot be easily distributed (e.g., tweets) and, in most cases, there are not publicly available collections of SM data. One of the few exceptions are the datasets developed under the CLEF eRisk challenge [18,26,27,28,29]. This is a series of shared tasks on early risk detection. The lab had a special orientation to psychological disorders and several datasets have SM data from depressed and control group individuals. We focus here on the eRisk 2017 and 2018 collections. The characteristics of these benchmarks are discussed next.

#### 4.2.1. Datasets: eRisk 2017 and eRisk 2018

The source of data used to build the eRisk collections was Reddit. This fits well with our Big Data platform, which ingests data from Reddit in real-time. Reddit has a large community of users and many of them have a large history of interactions on the platform. There is substantive content about psychological disorders, such as depression.

In creating these collections, a fundamental issue was how to determine what users have depression. As argued above, some researchers [4] have employed clinical depression surveys. But relying on self-reported questionnaires is tedious and requires to contact with every candidate. Furthermore, the amount of data obtained in this way is limited. Other authors, e.g., [7], designed automatic methods for identifying people diagnosed with depression. This was the approach followed in building the eRisk datasets. It is based on extracting self-expressions of diagnoses from the user’s SM submissions. These can be obtained by submitting certain queries to Reddit’s search service (for example, “I was diagnosed with depression”). The retrieved results, which are posts that contain some type of explicit mention of a diagnosis, were manually reviewed by the collection’s creators to ensure that the information given in the posts really looked genuine. The quality of the resulting collections is high and these benchmarks have been employed by dozens of research groups worldwide. Reddit posts tend to be long and explicit, and many of the retrieved posts come from the depression subreddit, a supportive space for users struggling with depression. Users suffering from depression often submit content to this subreddit and many of them are very explicit about their psychological diagnosis. The manual checks of the retrieved posts were strict. Expressions like “I am depressed”, “I have depression”, or “I think I have depression”, were not considered as an explicit declaration of a diagnosis. A user was included into the depression group only when there was an explicit and clear mention of a diagnosis (e.g., “Last week, I was diagnosed with depression by my doctor”). Following standard practice in this field, a control group was obtained by random sampling from the entire community of Reddit users. Furthermore, the creators of the eRisk datasets included into the control group some users who were active on the depression threads but had no depression (e.g., a doctor giving support to depressed users or a wife whose husband has depression). This type of users often discuss topics related to depression and incorporating them into the control group makes the collections more realistic (it is not only about distinguishing users based on the topic of their conversations). It is possible that the control group has some truly depressed user and it is possible that the depressed group has some user who has not depression (a user’s declaration about his diagnosis might be untrue). However, as argued in [18], the impact of such cases is expected to be negligible and other screening strategies (e.g., based on surveys) are not exempt from noise or false claims.

For each user, the eRisk collections contain up to 1000 posts and 1000 comments. The most active users have nearly 2000 submissions covering a large period of time. This includes posts or comments submitted to any subreddit. This allows to study the users’s natural language use (regardless of the topic of the conversation). Each user’s thread of submissions is organised in chronological order. In this way, the eRisk datasets allow to analyse not only language differences between the two groups of users, but also the evolution of the text written by each group. For each user, the post where he explicitly declared that he was diagnosed was removed from the collection. This removal prevents depression language classifiers from overfitting to the specific phrases (e.g., “diagnosed with”) used for creating the depression group.

Table 1 reports the main statistics of the eRisk 2017 and 2018 collections. Both datasets have two splits, a training set of users and a test set of users. Following the evaluation scheme proposed in the eRisk labs, we employed the train split to build a depression language classifier (using two-class supervised learning technology) and, next, the learned classifier was evaluated against the test examples. The performance of the test stage takes into account not only the accuracy of the decisions but also the delay (i.e., how many submissions per subject were needed to make the decisions). The main features of this evaluation scheme are explained next.

#### 4.2.2. Early Risk Evaluation Metrics

Given a language classifier (typically built from a set of training examples), and a set of test examples, which are chronologically ordered sets of SM submissions made by test users, an early risk detection challenge [18] consists of sequentially processing the available submissions and detect users at risk as soon as possible. For each user, his submissions have to be processed in the order they were created. Algorithms that effectively perform this task can be naturally incorporated into our Big Data platform, which monitors SM submissions in real time.

Let us consider a collection of SM submissions written by *k* different users: $U_{1},\ldots ,U_{k}$. Each user $U_{i}$ (i∈{1,…,k}) has $n_{i}$ submissions in the collection. The chronologically ordered sequence (or stream) of $U_{i}$’s submissions is denoted as: $S_{U_{i},1},S_{U_{i},2},\ldots ,S_{U_{i},n_{i}}$; where $S_{U_{i},1}$ is the oldest submission and $S_{U_{i},n_{i}}$ is the newest submission. Given *k* streams of submissions (one per user), the early risk detection task is defined as follows:

- An early risk detection system has to process the submissions following the order in which the submissions were created. At some point *p* ($p\in \{1,\ldots ,n_{i}\}$) the system has to emit a binary decision. This binary flag represents the system’s estimation about the user showing signs of depression.
- Positive cases need to be detected as soon as possible. However, there is a tradeoff between making more informed decisions (based on many submissions) and making early decisions (we act earlier but we base our decisions on fewer pieces of evidence).

Given golden truth judgements, which inform us about what users belong to the depressed group, we can compute standard classification metrics (e.g., F1, Precision or Recall) to evaluate the system’s estimations. However, these metrics are time-unaware and, therefore, the eRisk challenges complemented them with a new metric, ERDE, that rewards early alerts.

ERDE stands for Early Risk Detection Error and takes into account the correctness of the (binary) decision and the delay taken to emit the decision. The delay is a function that grows with the number of user submissions seen before giving the answer. Essentially, ERDE is an error measure that incorporates a penalty associated to the delay in detecting true positives. In the extreme case of detecting a true positive after hundreds of submissions, the error score assigned is equivalent to the error assigned to a false negative (i.e., late detection equals to no detection). Further details about ERDE can be found in [18].

#### 4.2.3. Validation of Depression Classifiers

In order to inject an effective depression classifier into CATENAE, we implemented several early detection methods and compared them with the collections and performance measures described above.

The training splits were used to create a depression language classifier, which was subsequently employed to support a number of early detection methods. In the training stage, each available user was represented with a single text, formed by concatenating all his writings. This single-document user representation was transformed into a vector of weights (one weight per word) using tf/idf [30]. This weighting method is a statistic that estimates how important a word is to a document based on a term frequency factor (which grows with the number of times the word appears in the document) and an inverse document frequency factor (which favours words that appear in fewer documents in the corpus). Given these vectorial user representations, we employed an effective two-class classification approach, based on Logistic Regression, to learn a depression language classifier from the available training users. More specifically, we performed L1 regularised Logistic Regression, which leads to sparse classifiers (uninformative features, i.e., uninformative words, are removed) whose computational requirements are prediction time are reduced. The classifier’s parameters were set by running 4-fold cross-validation on the training data. We finally proceeded to fix the optimal parameter setting and built the classifier from the whole training data.

Next, we tested different methods to sequentially process the chronology of texts written by the users in the test split. Some methods require the classifier built from the training data and some methods do not. The methods that we compared are:

- **Random**. This is a basic method that simply emits a random decision. It serves as a baseline for comparison. The decision is emitted right after seeing the first user submission. This method is fast but we expect it to perform poorly.
- **Depressed**. This is another fast (but naïve) baseline. It assigns the depression class to all users (after seeing the first submission). Observe that the alternative “non-depressed” baseline would not find any depressed user and, thus, it would score 0 on all performance metrics.
- **First *n***. This method consists of concatenating the oldest *n* user submissions, sending them to the depression language classifier, and making the prediction based on the output of the classifier. The delay is therefore always equal to *n*. A particular instance, where *n* is larger than the maximum number of user submissions, will be referred to as “All” (which sees all user submissions before making any prediction).
- **Incremental**. This method does not consider a fixed number of user submissions. Instead, it starts from the oldest user submission and incrementally builds a user representation. After each user submission the method passes the aggregated representation (submissions seeing so far) to the depression language classifier, and makes a “depressed” decision only if the depression language classifier is confident enough (the depression language classifier outputs a probability score that represents its confidence on the user being depressed; we tested the following confidence thresholds: 0.5, 0.75 and 0.9). Observe that this method can emit the decision at any point (for example, right after the first user submission or right after the last submission). If the stream of user submissions gets exhausted then the method concludes assigning “non-depressed”.

Table 2 reports the results of these experiments. The comparison includes three standard metrics, namely:

(1) $$\begin{array}{ccc} Precision(P) & = & \frac{TP}{TP+FP} \end{array}$$

(2) $$\begin{array}{ccc} Recall(P) & = & \frac{TP}{TP+FN} \end{array}$$

(3) $$\begin{array}{ccc} F1 & = & \frac{2\cdot P\cdot R}{P+R} \end{array}$$

where TP are the true positives (depression cases correctly labelled by the system), FP are the false positives (non-depression cases labelled as depressed by the system), and FN are the false negatives (depression cases labelled as non-depressed by the system).

Two configurations of the ERDE measure were tested, $ERDE_{5}$ and $ERDE_{50}$. With $ERDE_{5}$, the penalty for late detection of true positives grows quickly after 5 submissions. $ERDE_{50}$ is a less stringent version of the measure where the penalty grows after 50 submissions. Note that, unlike *P*, *R* and F1, ERDE is an error measure and, therefore, the lower the better.

It is not surprising that the random method and the depressed method are the worst performers. Of course, the depressed method yields perfect recall but its precision and ERDE scores are really poor. The fixed-length methods (First) score reasonably well in terms of F1 but their ERDE performance scores show that they are slow at detecting depression cases. In one of the collections, the All method, which consists of analysing all user’s submissions, yields the highest F1. However, this method is suboptimal in terms of early risk detection. The incremental variants are the most consistent methods. They can make quick decisions and still yield competitive F1 scores. The incremental method with threshold equal to 0.5 is the most solid approach. It has a good balance between correctness and delay (yields the lowest ERDE scores and has competitive F1 scores). We therefore decided to adopt this method for our Big Data platform.

Although the overall performance levels are modest, these classification methods are not meant to be used for taking automatic decisions about particular individuals. As shown below, our platform is oriented to support expert users in identifying risks of depression. The capability of the platform to ingest massive amounts of user data and the ability of the classifier to focus on signs of depression make a good combination to screen SM data.

Next, we explain how to deploy this classifier into our streaming architecture. To this aim, we incrementally analyse the stream of texts written by each SM user and estimate the onset of signs of depression. The system incrementally builds a representation of each user, passes the user representation to the depression language classifier, and, if the classifier is confident enough, the system *fires an alert*. The system is oriented to early risk detection and, thus, it alerts as soon as it finds evidence of a potential risk.

### 4.3. Pipeline Architecture

The classification tasks are handled by modules that will be referred to as predictor microservices. The resulting architecture naturally scales up. If a preprocessing microservice is slower than the predictor then we can augment the number of instances associated to the preprocessing stage. This stream-based approach is highly efficient and more convenient than a batch processing approach for generating alerts as soon as possible.

**Figure 4:**
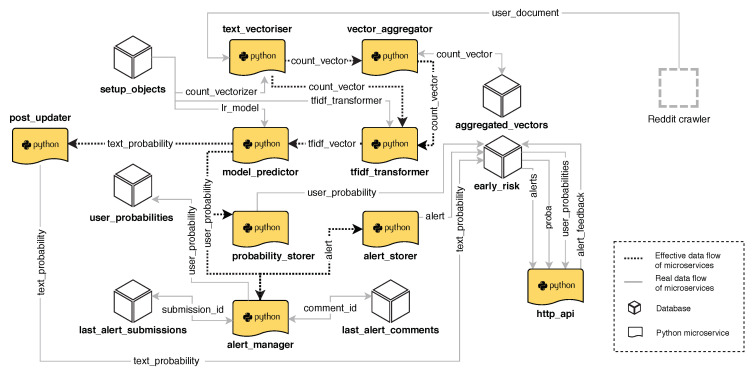
Architecture that supports early detection of signs of depression.

The prediction stage requires several microservices, which are sketched in Figure 4:

- A **Text Vectorisation** module transforms any input text into a vector of counts (number of occurrences of each term in the vocabulary).
- An **Aggregator** receives the new vectors of counts (coming from new posts or comments) and is responsible for producing an accumulated vector for each user. This vector represents all posts and comments submitted by the corresponding user. The aggregator constantly merges the current vector of counts with vectors coming from any new post or comment.
- The **Tf–idf Transformation** module takes the accumulated vectors, which store term frequency counts, and produce a normalised tf/idf representation. To this aim, inverse document frequency (idf) statistics are obtained from offline data (More specifically, corpus statistics are obtained from the depression and language test collection [18]).
- **Model Prediction** module, which estimates the onset of signs of depression from the normalised tf/idf representations. It employs the L1-penalised logistic regression classifier and produces a probability estimate for each user. This probability value is stored by the next microservice.
- **Probability Storer** module, which receives and stores the estimated probabilities, and the **Alert manager** module, which also receives the estimated probabilities and is responsible for emitting alerts when probabilities are greater than a given threshold.

The storage and access operations are fast because we utilise Aerospike to store the accumulated vectors and other required objects, such as the representation of the vocabulary, the classifier and the tf/idf transformer. In our architecture, microservice initialisations and vector updates happen in a very efficient way.

### 4.4. Social Media Depression Screening Tool

Using the Big Data technologies described above, we developed a prototype screening tool that constantly analyses SM data and emits real-time alerts about the appearance of signs of risk. Such a tool could be used in the future by public health experts. For example, the tool helps to analyse specific groups or SM communities, to understand the onset and evolution of psychological disorders, to learn about the main symptoms or topics discussed by people suffering from depression, etc. It can also help researchers to build new datasets and test collections from SM data. As a matter of fact, we are currently collaborating with psychologists in order to further exploit this tool to build new collections for different disorders. As argued above, the platform is modular and adaptable to multiple monitoring tasks and by incorporating other types of user classifiers we could support different SM intelligence tasks and users (e.g., it could help SM moderators to early identify inappropriate behaviours).

**Figure 5:**
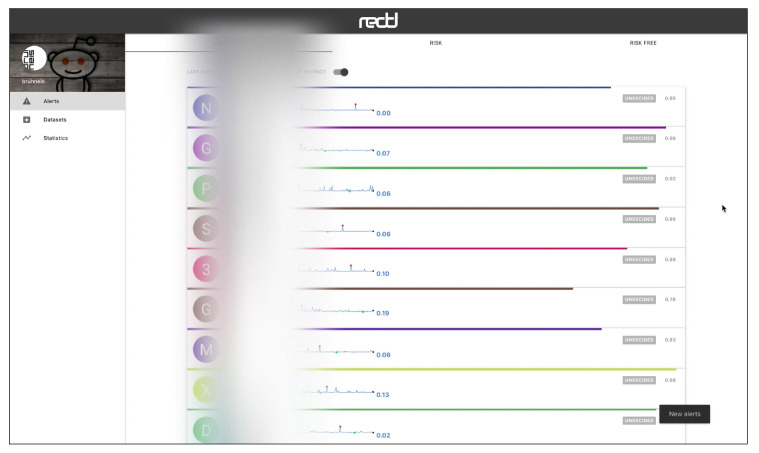
View of real-time alerts of signs of depression (real usernames have been obfuscated).

**Figure 6:**
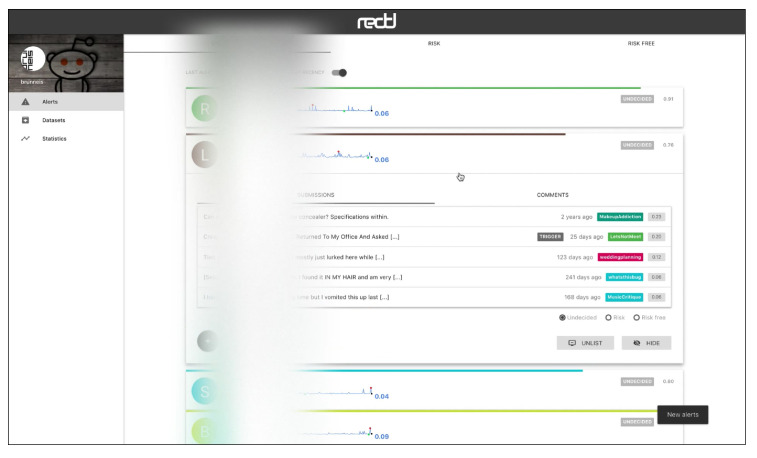
View to inspect the user’s submissions that generated a given alert (user data have been obfuscated).

**Figure 7:**
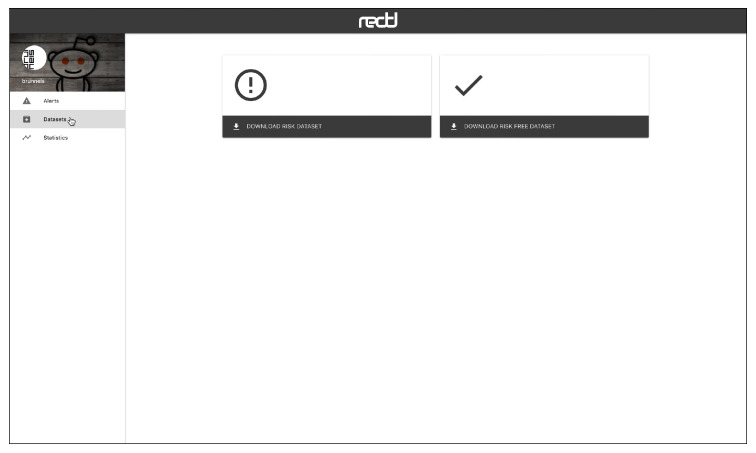
View to download the extracted dataset.

**Figure 8:**
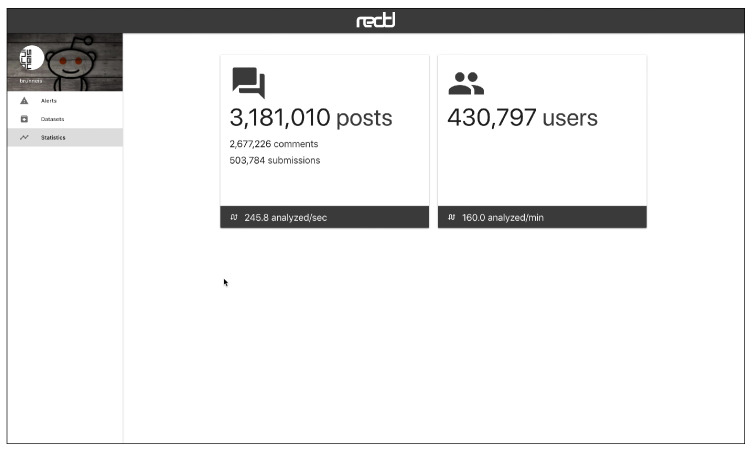
Statistics view.

The web interface has three main views, which are connected to a HTTP API. Figure 5 shows the “Alerts” view, where the user can inspect the alerts emitted by the system in real time. The alerts can be ordered by recency or priority (where the users whose probability of depression is higher are shown first). Each alert corresponds to one user and the tools presents the user’s posts and comments. The individual submissions are ordered by decreasing probability and the expert can review the user’s history of submissions. After inspection, the expert can label the alert as a true positive (risk) or false positive (non-risk). In this way, the tool facilitates the construction of new SM datasets and benchmarks. When an alert is fired, the tool also plots a line chart that represents the temporal evolution of the user (see Figure 6). Each point in the plot represents the estimated probability of depression obtained at a given point of time. These probability values are generated at the time points where the user made new submissions (the new post or comment is employed to update the user’s representation and, next, the user classifier produces a new probability estimate). The alerts are organised into three tabs in the view. The default tab contains the untagged alerts, while the tagged alerts are organised into a true positives tab and a false positives tag. The “Datasets” view allows the expert to download the true positives, the false positives or all users (see Figure 7). The “Statistics” view reports basic real-time information about the screening tool (total number of post or comments processed, number of users analysed, and processing speed (see Figure 8)).

### 4.5. Performance Analysis

We deployed the architecture described above in a well-known cloud computing service, Amazon Web Services (AWS). AWS supports a wide range of virtual machines in its EC2 infrastructure. We utilised c5.4xlarge instances that have the following characteristics:

- CPU: Intel(R) Xeon(R) Platinum 8124M CPU @ 3.00 GHz (16 assigned virtual cores).
- Memory: 32 GB.
- Storage: 100 GB SSD General Purpose volumes (EBS).
- Dedicated Bandwidth: up to 2.250 Mbps for EBS storage

The general network performance is around 5 Gbps. In this series of experiments, we considered that processing was performed in real time when all texts were classified within seconds (with no growing queue of pending texts). In order to remove bottlenecks, we determined experimentally the proportion of each type of microservice instances in each execution graph. For crawling purposes, the User Content Crawling module was the only module that required scaling. For classification purposes, the most demanding stages are Text Vectorisation, Tf-idf Transformation, Vector Aggregation and Model Prediction and we assigned the proportions 2-4-4-1 (respectively) in order to balance the workload.

The parallelism score refers to the number of times that the selected microservices were replicated. For instance, a parallelism score equal to 4 in the classification execution graph would assign 8x Text Vectorisation instance, 16x Tf-idf Transformation instances, 16x Vector Aggregation instances and 5x Model Prediction instances.

**Figure 9:**
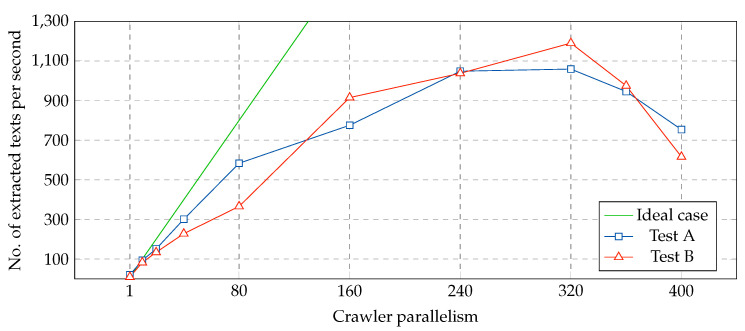
Performance of the crawler with varying parallelism settings (one node).

Figure 9 reports the number of extracted texts per second for two crawling experiments performed on Reddit in different days. A single virtual machine was launched and crawler parallelism was tested from 1 to 400. Each configuration was executed during five minutes. Reddit’s workload and response times varied over the experiments and, thus, the two tests show slightly different patterns. With higher response times, the optimal number of instances of the crawler is higher. When the parallelism score was greater than 320, performance starts to degrade for both experiments.

**Figure 10:**
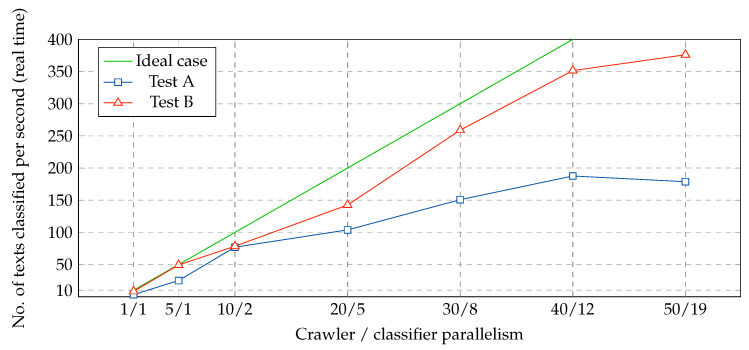
Parallelism level required to achieve real-time processing in one node.

Figure 10 reports the results of two experiments run with the classifier and the crawler during different days. To ensure real-time processing of the submissions, crawler parallelism was evaluated from 1 to 50 (with crawler parallelism scores higher than 50, real-time processing was not achieved) and the parallelism of the classifier was directly set based on the crawler’s parallelism score. Each configuration was executed during five minutes. This experiment was repeated for Test B with the same parallelism settings. The results were highly satisfactory (higher efficiency of the crawler compared with Test A, while still maintaining real-time processing).

**Figure 11:**
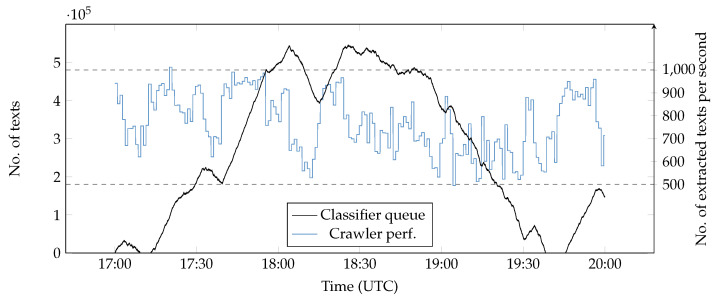
Three-hour experiment (using 3 nodes). The parallelism levels (crawler/classifier) were 170/0, 0/50 and 0/50.

In a final experiment, we configured a three-node cluster using Apache Kafka and ran an experiment for three hours (see Figure 11). There were large fluctuations on crawling capacity (between 500 and 1000 extracted submissions per second). As a consequence, the size of the queue of pending (unclassified) aggregated vectors oscillated substantially. This suggested that a fixed parallelism in both topologies (or even a fixed number of nodes) is not an optimal choice. In order to perform better, the system should scale up and down automatically and avoid wasting resources. In the future, we will work on designing an execution graph controller that automatically adapts the parallelism levels to the current workload.

## 5. Connection with External Systems

The library utilises Kafka and this choice facilitates the connection between our execution graph and external systems. We can set up *producer* connections (where the external source emits data to the execution graph) or *consumer* connections (where the external source consumes data from the execution graph). To this aim, we only need to incorporate a Kafka consumer or producer into the external system.

**Figure 12:**
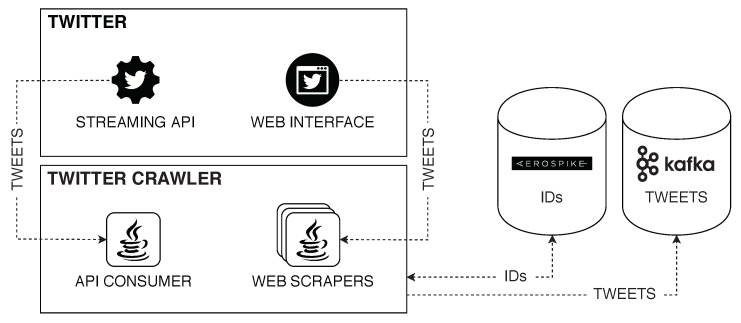
POLYPUSmathsizesmall’ Twitter crawler.

Frameworks such as POLYPUSmathsizesmall [31] can be easily connected to our library. POLYPUSmathsizesmall [31] is a modular platform that provides functionalities such as data extraction from SM, real-time sentiment analysis, and distributed non-relational storage (just to name a few). The core modules of POLYPUSmathsizesmall are not written in Python but POLYPUSmathsizesmall can naturally act as a data source for CATENAE. Figure 12 illustrates how to include a Kafka producer that connects POLYPUSmathsizesmall with CATENAE. The resulting Twitter crawler sends tweets to the input of a CATENAE execution graph. This connects a module written in Java (from POLYPUSmathsizesmall) and an execution graph written in Python (from CATENAE) and, thus, data should be in the form of basic types.

## 6. Related Work

### 6.1. Social Media Intelligence

In recent years, many studies have explored the potential of SM to predict the onset of psychological problems. The interactions between SM activity and psychological traces have been the focus of attention of popular evaluation campaigns, such as the CLPsych shared tasks [32,33], organised within the Computational Linguistics and Clinical Psychology Workshop, or the eRisk tasks [26,27,28], oriented to early detection of signs of psychological problems and organised within the Conference and Labs of the Evaluation Forum (CLEF). These shared tasks have instigated research on how different search and language technologies (e.g., Text Classification, Information Retrieval and Text Mining) can support health intelligence. To this aim, the organisers of these challenges collected data from SM (e.g., CLPysch collected posts in online peer-support fora and eRisk collected SM submissions –posts and comments– from Reddit) and asked participants to perform a certain task. For example, CLPsych asked participants to distinguish between the posts written by individuals who suffer from a certain mental disorder from the posts written by individuals who do not suffer from such disorder. Rather than a two-class classification problem, the eRisk shared tasks focused on early identification of signs of psychological problems and, thus, the participants had not only to make accurate predictions about the presence of psychological problems but to do it quickly (e.g., after seeing few pieces of evidence in the form of SM submissions).

Over the years, many research efforts have been focused on SM and psychological disorders. The team led by De Choudhury has been very active on analysing SM and mental health [4,6,22,34]. In [4], they explored the potential to use Twitter feeds to detect and diagnose depression episodes. To this aim, they employed existing tools such as Linguistic Inquiry and Word Count (LIWC) [35] and well-known medical questionnaires, such as the Center for Epidemiologic Studies Depression Scale (CES-D). A discussion on the role of SM and its potential in developing automatic screening tools can be found in [6]. In [22], it was analysed how to employ SM data as a measurement tool of depression in populations. The study suggests that evidence obtained from SM can complement traditional survey techniques. Furthermore, the results show that a SM-based depression index confirms several psychiatric findings and correlates with statistics reported by health institutions.

Coppersmith and colleagues [36] considered Post Traumatic Stress Disorder (PTSD) and demonstrated the utility of Twitter data in building automatic classifiers that exploit differences in language use between PTSD and non-PTSD individuals. In a similar vein, the same team of researchers showed in [7] that Twitter can be a valuable source of evidence to enhance the data about PTSD, depression, bipolar disorder and seasonal affective disorder available to clinicians. Cohan and colleagues [37] explored how to create large-scale, labelled datasets related to mental health. To this aim, they worked with Reddit data and employed patterns to identify self-reported diagnoses (users who publicly express that they have been diagnosed with a certain health condition). The resulting dataset was used to compare different text classification methods.

Other sources, such as Tumblr, were used to study behavioural characteristics of users suffering from certain eating disorders [38]. The detection and characterisation of eating-disorder (and even pro-eating disorder groups) communities was also explored in [39,40].

Benton and colleagues [41] recently discussed a crucial issue: ethics and SM health research. The main outcome of their study is a set of practical guidelines for researchers working with SM data for health-related research. A recent analysis on ethics for online risk detection Artificial Intelligence (AI) Systems was done by Razi and colleagues [42]. They proposed a number of ethical considerations related to systems that attempt to keep adolescents safe by providing accurate AI-based services to adolescents and their parents.

The studies described above are really valuable, as they contributed significantly to shape the foundations of this area of research. However, the experiments were performed using specific data samples extracted from SM and, to the best of our knowledge, none of these studies explored how to implement this type of health-related predictive technology in real time. This was precisely the main aim of our paper.

The next subsection discusses other class of studies, centred on Big Data technologies, that are also related to our research.

### 6.2. Big Data

MapReduce [43] was introduced by Google as a programming paradigm that supports the generation and processing of huge data sets on a large set of computing nodes. Following MapReduce, the execution of a program execution is split into two stages: *map* and *reduce*. Each MapReduce computation receives as an input and outputs a list of pairs (key,value). The users of a MapReduce platform only need to provide their custom implementation of the functions map and reduce. At the map stage, map workers take their input and generate a set of intermediate key-value pairs. This sequence is stored on an intermediate storage (e.g., memory buffers or files). The reduce function takes the intermediate key-value pairs and produce a final output. The map workers provide data parallelism (e.g., they work in parallel on different segments of the input data), while the reduce workers perform parallel reduction (aggregate or combine results). Currently, multiple frameworks support the MapReduce programming model.

Apache Spark [44] provides a processing model that is more flexible than Hadoop MapReduce [45]. Spark supports arbitrary workflows where custom processing phases can be defined and it can incorporate operations beyond *map* and *reduce*. Spark is written in Scala (JVM language) and supports Python. However, the execution of code written in non-JVM languages is less efficient because Spark does not natively execute non-JVM code. Our approach, based on CATENAE, can naturally incorporate new or existing modules with fewer modifications on its code. Custom data structures are not required and, if the modules were already distributed in a pipeline with multiple scripts, the transition is more natural. Furthermore, Spark does not naturally support Python dependency management (because the required libraries have to be available at all the nodes of the cluster). In contrast, CATENAE handles dependencies by incorporating a Docker image for each module. This image includes the Python interpreter and contains the dependencies required by the module. In this way, the context and performance of a Python module executed in the platform is the same as the context and performance of the same module when executed locally within a Docker container. In Spark, data cannot go back to early phases because the workflows have to be directed acyclic graphs. CATENAE focuses on real-time, where cycles naturally occur (e.g., a module that checks a time condition in order to let the data flow).

The existing processing technologies discussed above need a cluster manager that executes the application (e.g., Apache Hadoop YARN [46], Apache Mesos [47]). CATENAE, in contrast, only needs Docker, a *de facto standard* framework for containers. The functionality of native clustering is supplied by Docker Swarm.

Apache Storm [48] is a framework capable of processing streaming data in real time. Storm requires *topologies*, which defined as computational graphs (workflows) where every node represents an individual processing task. Edges correspond to the data flowing between nodes. Data is exchanged in the form of *tuples* (ordered lists of values, where each value has a name assigned). The nodes exchange *streams*, which are non delimited sequences of tuples. Every node listens to one or multiple streams as input. In Storm terminology, the name *spouts* refers to the sources of a stream within an execution graph. *Spouts* often read data from external sources. *Bolts* are the consumers, as they perform computations and transformations on the data. Bolts can emit zero, one or multiple tuples to the output streams. When an execution graph begins, it waits for new data to be processed. Storm clusters have two classes of nodes: master (Nimbus) and workers (supervisors). Storm employs Apache Thrift [49] and, thus, topologies could be defined and submitted using any programming language. However, non-JVM languages interact with Storm through a JSON-based protocol. In this way, Python dependencies have to be installed in the cluster nodes and management is inefficient because different topologies typically need different versions of the libraries or packages.

Kafka is a distributed message broker for low-latency, high-throughput that handles real-time data feeds. In CATENAE, we employ Kafka as a mechanism to distribute messages between the modules of an execution graph. Using Kafka directly would require to handle Kafka producers and consumers for each execution node. Kafka Streams is an official Kafka library for building real-time topologies. However, Kafka Streams is only available for Java and Scala.

CATENAE topologies can be deployed using Docker [50] containers, leading to benefits in terms of virtualisation (e.g., isolation, portability, flexibility, or agility). Docker does not penalise I/O performance in a substantial way and makes good use of resource isolation of the Linux kernel (independent containers can run on the same host). Containers are constructed with stacked layers and supply a virtual environment with their own space of networks and processes.

## 7. Ethical Issues

This paper proposes Big Data technologies that can support socially-valuable surveillance tasks. However, the adoption of screening tools for Public Health surveillance needs to consider proper ethical boundaries. As argued by Olteanu and colleagues [51], social data can enable better decision-making in multiple fields but there is a broad variety of menaces. Analysing SM data needs to be done with rigour. Otherwise, unexpected consequences may arise.

The platform developed here is not meant to be use as a standalone tool that takes decisions on its own. Instead, it is designed to support the decision of human experts. These experts need to be cautious about social data use. For example, we need to be critical about how reflective SM user behaviour is of the real-world phenomenon being studied (e.g., how reflective SM submissions are of the psychological state of a given individual?). Another issue affects the samples and SM sources analysed. If the expert monitors a single SM site (e.g., Twitter) then she needs to consider how extrapolated to other media the findings are.

This paper has focused on the technological challenges involved in designing efficient tools able to analyse SM data in real-time. In order to deploy this technology for health surveillance, a full analysis of ethical implications and social consequences needs to be done. Some authors have already suggested the potential of online screening for signs of depression. For example, Neuman and colleagues [17] argued that the high prevalence of depression in the modern society puts severe constraints on the ability of public systems to provide adequate diagnostics and treatments. With proper permissions and rigorous ethical guidelines, public health agencies might benefit from the ability of Big Data tools to analyse thousands of texts and identify possible signs of risk. Furthermore, this analysis and risk detection methods could be employed at different levels (individuals, communities, regions or even countries). Note also that interventions do not need to target particular individuals. Instead, the SM analysis could be oriented to design pre-emptive measures. For example, the appearance of certain signs of depression (e.g., increasing dissatisfaction, critical of self or weight loss) can lead to public policies and campaigns oriented to overcome these problems.

## 8. Conclusions

In this paper we presented the main features of CATENAE, a Python library that facilitates the construction of scalable real-time streaming applications. Our technological solution can naturally support Social Media Intelligence tasks. As a matter of fact, we showed here the potential of CATENAE for analysing Social Media streams in order to support the detection of early signs related to depression.

The proposed architecture is flexible and rapidly digests thousands of SM submissions. In the execution graph, data can flow following any direction (even backwards). Dependency issues (e.g., problems with library versions or lack of packages on the cluster) are avoided through the use of Dockers. Scalability is achieved by launching more containers for the same module. Isolation also allows us to run scripts written with different versions of Python. Basic input data prioritisation politics are naturally handled and they need not to be manually coded. Serialisation and deserialisation of Python objects is made automatically.

As future work, we plan to extend the use of the platform to other application domains (beyond the analysis of depression). We also plan to validate the functionality of the system with expert users (e.g., psychologists or clinicians). Another interesting line of work is multi-linguality. In this paper we validated our classifiers using samples of SM submissions written in English. However, the technology is flexible and could be adapted to other languages. In this respect, we will study how to exploit recent advances in supervised and unsupervised cross-lingual word representations [52].

From a technological point of view, we also consider some possible lines of future work. For example, we plan to implement new queue handlers (and, possibly, custom handlers). We only depend on Docker and, thus, deploying topologies will be done in an automatic way. Another enhancement would be to deploy a disposable Kafka cluster with the execution graph. This would allow the administrator of the platform to scale up and down the execution graph. Real-time applications often suffer dramatic changes in their workload and being able to automate the scaling is an attractive feature.

## Figures and Tables

**Table 1:** Main statistics of the eRisk 2017 and 2018 collections.

	**eRisk 2017**			
	Train Split		Test Split	
	*Depressed*	*Control*	*Depressed*	*Control*
Num. users	83	403	52	349
Num. submissions (posts+comments)	30,851	264,172	18,706	217,665
Num. of user submissions (avg)	371.7	655.5	359.7	623.7
Num. of days from first to last submission (avg)	572.7	626.6	608.31	623.2
Num. words per submission (avg)	27.6	21.3	26.9	22.5
	**eRisk 2018**			
	Train Split		Test Split	
	*Depressed*	*Control*	*Depressed*	*Control*
Num. users	135	752	79	741
Num. submissions (posts+comments)	49,557	481,837	40,665	504,523
Num. of user submissions (avg)	367.1	640.7	514.7	680.9
Num. of days from first to last submission (avg)	586.43	625.0	786.9	702.5
Num. words per submission (avg)	27.4	21.8	27.6	23.7

**Table 2:** Comparison of classifiers. Classification and early detection metrics.

	**eRisk 2017**				
	F1	P	R	$ERDE_{5}$	$ERDE_{50}$
Random	0.176	0.110	0.442	0.133	0.133
Depressed	0.230	0.130	1.000	0.113	0.113
First 10	0.254	0.474	0.173	0.113	0.112
First 100	0.574	0.592	0.558	0.073	0.070
First 500	0.571	0.533	0.615	0.069	0.066
All	0.584	0.541	0.635	0.067	0.064
Incremental 0.50	0.497	0.376	**0.731**	**0.064**	**0.059**
Incremental 0.75	**0.600**	0.625	0.577	0.068	0.065
Incremental 0.90	0.513	**0.769**	0.385	0.086	0.084
	**eRisk 2018**				
	F1	P	R	$ERDE_{5}$	$ERDE_{50}$
Random	0.169	0.100	0.532	0.089	0.089
Depressed	0.176	0.096	1.000	0.087	0.087
First 10	0.263	0.429	0.190	0.082	0.082
First 100	0.563	0.556	0.570	0.051	0.050
First 500	0.551	0.495	0.620	0.048	0.047
All	**0.565**	0.510	0.633	0.047	0.046
Incremental 0.50	0.464	0.348	**0.696**	**0.046**	**0.043**
Incremental 0.75	0.530	0.506	0.557	0.052	0.050
Incremental 0.90	0.432	**0.587**	0.342	0.068	0.067

For each measure the highest performance (F1, P, R) or lowest error ($ERDE_{5}$, $ERDE_{50}$) is marked in bold.
